# Significant reduction in depressive symptoms among patients with moderately-severe to severe depressive symptoms after participation in a therapist-supported, evidence-based mobile health program delivered via a smartphone app

**DOI:** 10.1016/j.invent.2021.100408

**Published:** 2021-06-17

**Authors:** Valerie L. Forman-Hoffman, Benjamin W. Nelson, Kristian Ranta, Albert Nazander, Outi Hilgert, Joao de Quevedo

**Affiliations:** a Meru Health Inc., San Mateo, CA, USA; b Translational Psychiatry Program, Faillace Department of Psychiatry and Behavioral Sciences, McGovern Medical School, The University of Texas Health Science Center at Houston (UTHealth), Houston, TX, USA; c Center of Excellence on Mood Disorders, Faillace Department of Psychiatry and Behavioral Sciences, McGovern Medical School, The University of Texas Health Science Center at Houston (UTHealth), Houston, TX, USA; d Neuroscience Graduate Program, The University of Texas MD Anderson Cancer Center UTHealth Graduate School of Biomedical Sciences, Houston, TX, USA; e Translational Psychiatry Laboratory, Graduate Program in Health Sciences, University of Southern Santa Catarina (UNESC), Criciúma, SC, Brazil; f University of North Carolina at Chapel Hill, Department of Psychology and Neuroscience, Chapel Hill, NC, USA

**Keywords:** ECT, electroconvulsive therapy, HRVB, heart rate variability biofeedback, ITT, intention-to-treat, MHP, Meru Health Program, PHQ-9, Patient Health Questionnaire-9 item survey, TMS, transcranial magnetic stimulation, Depression, Mobile health interventions, Digital health interventions, Severe depression

## Abstract

Depression is a debilitating disorder associated with poor health outcomes, including increased comorbidity and early mortality. Despite the advent of new digital health interventions, few have been tested among patients with more severe forms of depression. As such, in an intent-to-treat study we examined whether 218 patients with at least moderately severe depressive symptoms (PHQ-9 ≥ 15) experienced significant reductions in depressive symptoms after participation in a therapist-supported, evidence-based mobile health (mHealth) program, Meru Health Program (MHP). Patients with moderately severe and severe depressive symptoms at pre-program assessment experienced significant decreases in depressive symptoms at end-of treatment (mean [standard deviation] PHQ-9 reduction = 8.30 [5.03], Hedges' *g* = 1.64, 95% CI [1.44, 1.85]). Also, 34% of patients with at least moderately severe depressive symptoms at baseline and 29.9% of patients with severe depressive symptoms (PHQ-9 ≥ 20) at baseline responded to the intervention at end-of-treatment, defined as experiencing ≥50% reduction in PHQ-9 score and a post-program PHQ-9 score lower than 10. Limitations include use lack of a control group and no clinical diagnostic information. Future randomized trials are warranted to test the MHP as a scalable solution for patients with more severe depressive symptoms.

## Highlights

• 218 patients with severe depression participated in a mHealth intervention.
• Patients had significant decreases in depression (Hedges' *g* = 1.64).
• Improvements were maintained at 3- and 6-months post-program.
• Nearly one-third of patients responded to the intervention at end-of-treatment.
• Future randomized trials are needed to test the intervention as a scalable solution.

## 1 Introduction

Depression is a debilitating global public health burden. Over 7% of U.S. adults have experienced major depression in the past 12 months (nearly 18 million adults; SAMHSA, 2019) with about two-thirds of whom have disorders categorized as severe. Depression has profound societal costs associated with its high rates of comorbid mental and physical health disorders and mortality, functional impairment, and decreased quality of life (Angermeyer et al., 2002; Katon, 2003; Wulsin et al., 1999). In addition, economic consequences of depression include high rates of healthcare utilization coupled with absenteeism and decreased work productivity (Stewart et al., 2003; Unützer et al., 2009). Because patients with severe forms of depression are more likely to experience poor outcomes than those with mild forms of depression (Katon et al., 2010; Kraus et al., 2019; Stewart et al., 1983), finding an acceptable treatment that works for patients with severe depression is particularly critical. For example, some evidence shows that patients with more than moderately severe depressive symptoms are more likely to need medication, be resistant to treatments, take longer to recover, relapse after recovery, self-harm, and/or attempt suicide than patients with more mild depressive symptoms (Nemeroff, 2007). Thus, patients with severe depression may need to try several interventions before finding one that works.

Some clinicians classify depression according to the severity of symptoms and associated impairment to guide the selection of appropriate treatment (Zimmerman et al., 2012). Clinical guidelines for depression tend to have differential recommendations based on depression severity. For example, several recommendation guidelines suggest both antidepressants and therapy should be used to treat severe depression (Gelenberg et al., 2010; Health UK, 2010), in part because several trials have shown that the efficacy of antidepressants varies based on symptom severity (Fournier et al., 2010; Kirsch et al., 2008). Furthermore, the long lag between the initiation of some types of medication therapy and therapeutic effect, which can take 5–7 weeks on average (Harmer et al., 2009), suggests the need, in some patients, for non-medication interventions or co-interventions, at least in the interim. Psychotherapy alone, however, is often not feasible for some patients experiencing symptoms of depression that impair cognition or reasoning in the acute phase of their depression so it is not recommended as monotherapy for patients with severe suicidality or psychotic symptoms (Excellence and Britain, 2004; Gelenberg et al., 2010). In addition, the use of medication therapy is not preferred by some patients who experience side effects that may attenuate or even negate medication benefits (McHugh et al., 2013; Van Schaik et al., 2004), particularly if they cause the patient to discontinue medication. Thus, choosing treatment for individuals with depression requires the clinician and the patient to carefully consider the research evidence on efficacy weighed against the potential side effect profile and likelihood of patient adherence to the protocol (Gartlehner et al., 2008).

Several treatments typically reserved for patients with more severe or treatment-resistant forms of depression include transcranial magnetic stimulation (TMS), electroconvulsive therapy (ECT), vagus nerve stimulation, deep brain stimulation, and fast-acting temporary relief medications such as ketamine (Appleby et al., 2007; Chen et al., 2013; Daban et al., 2008; Kishimoto et al., 2016; Pagnin et al., 2008). Use is not widespread, however, possibly due to each of these treatment options having particularly notable side effects, acceptability, or access issues (Nemeroff, 2007). For example, although ECT generally is fast-acting and considered to be the most effective treatment for severe depression (Sackeim et al., 2007), factors such as intensity of the treatment, long administration time, risk of associated side effects including long-term cognitive problems, need for anaesthesia that requires hospitalization, and long recovery times after the treatment might decrease the likelihood that patients and providers choose it. Thus, ECT is primarily recommended only in instances of severe depression that either does not respond to other interventions or that requires fast-acting treatment in order to reduce suicidality (Sackeim et al., 2007; Sonawalla and Fava, 2001).

As such, alternative treatments acceptable to patients with severe forms of depression where benefits outweigh the potential harms are urgently needed. Several digital health interventions have shown promise in treating depression, particularly those that include clinician support (Firth et al., 2017; Weisel et al., 2019). Few, however, have been tested among patients with severe depression, partially due, perhaps, to the common perception that digital health interventions are more suitable to treating milder forms of depression (Topooco et al., 2017). This belief lies in contrast to some evidence indicating that patients with severe depression might experience even greater benefits in response to resource-limited interventions than patients with more mild forms of the disorder (Bower et al., 2013). The current intent-to-treat (ITT) study investigated some of these claims using real-world data. In this secondary data analysis of existing clinical data, we sought to study the changes in depressive symptom outcomes among patients with at least moderately severe depressive symptoms (PHQ-9 ≥ 15) who participated in a therapist-supported, evidence-based digital health intervention delivered via a smartphone application during 2020.

## 2 Materials and methods

### 2.1 Research design

We examined existing clinical data collected from patients treated with the Meru Health Program (MHP) before the start of treatment, every 2 weeks during the 12-week treatment period, and at 3- and 6- months post-intervention. Although prior studies utilizing real world Meru Health data have been published, this is the first time we report findings on participants who entered the program during 2020 who were solely treated with the 12-week MHP that incorporates heart rate variability biofeedback (HRVB). Further details about the sample and prior publications are described below.

Institutional review board exemption for this analysis was granted by Pearl Institutional Review Board for analyses of previously collected and de-identified data. Data collected as part of care provided by the MHP is stored in Health Insurance Portability and Accountability Act-compliant electronic medical records that includes protected health information. All data is encrypted in transit, and at rest.

### 2.2 Participants

Meru Health operates a professional corporation in California and Florida and has been treating patients since 2018.The present study included patients treated at the Meru Health Online Clinic during 2020. All patients who started the program on or after January 1, 2020 and on or before August 21, 2020 were included in analyses. We used November 13, 2020 as a cut-off to allow for inclusion of patients able to complete the 12-week treatment program and have a 6-month follow-up in our dataset.

Participants entered the Meru Health Program via referrals from employee assistance programs or via their healthcare providers. All enrolled patients signed informed consent to participate and to have their collected and deidentified data used for research purposes. Inclusion/exclusion criteria of the MHP required patients to have at least mild levels of depression, anxiety, or burnout, own a smartphone, and not have an active substance use disorder, severe active suicidal ideation with a specific plan, severe active self-harm, or a history of psychosis or mania.

For the present study analyses, only patients with moderately severe to severe depressive symptoms at baseline, defined as having a Patient Health Questionnaire-9 item (PHQ-9) score of 15 or higher at the pre-MHP (baseline) assessment, were analysed. In general, PHQ-9 scores in the 15–19 range indicate moderately severe levels of depressive symptoms, while PHQ-9 scores of 20 or greater typically represent severe forms of depressive symptomatology (Kroenke et al., 2001). At the time of data analysis for this study, 218 MHP patients started the MHP with a baseline PHQ-9 scores of 15 or higher; 141 started with scores between 15 and 19 and 77 started with scores of 20 or higher.

### 2.3 Intervention

The MHP has been described in detail in prior publications (Economides et al., 2019b; Goldin et al., 2019). In sum, the MHP is comprised of several different evidence-based components delivered via a smartphone app. The program is self-guided but incorporates a continuous care model that includes daily interaction with a dedicated, licensed clinical therapist, with a medical doctor and psychiatrist available for consultation as needed. The original program had contained components of Cognitive Behavioral Therapy (Beck, 1979), Behavioral Activation Therapy (Jacobson et al., 2001), Mindfulness-Based Stress Reduction (Kabat-Zinn, 2009), and Mindfulness-Based Cognitive Therapy (Morgan, 2003) and lasted 8 weeks in duration. Over time, the MHP has expanded to 12 weeks of content with the addition of sleep therapy (Carney et al., 2017) and nutritional psychiatry components (Sarris et al., 2015). In addition, heart rate variability biofeedback (HRVB) was added to some intermediary versions of the program and maintained in current versions as of December 2019. To mitigate changes in content, we chose to examine patients entering the MHP during 2020, when the only available version of the program was the 12-week program that included HRVB.

Prior to the start of the MHP, participants have an intake video call with the therapist and then are trained on how to use the app, including how to participate in the anonymous group interaction and how to communicate with their assigned therapist via chat or, when specifically requested, phone or video calls. Each week of the program begins with an introductory video that gives the patient an overview of the topics covered that week. Watching the introductory video unlocks all content for that particular treatment week. On weeks when content is unlocked, patients are prompted to complete various practices on a daily basis, mostly mindfulness mediation and HRVB practices as well as short CBT exercises and journal prompts. Daily content and practices range from 5 to 15 min, except for the first day of each week, in which the weekly psychoeducation video lessons can extend the content to a maximum of 25 min.

A licensed therapist (employed by Meru Health) provides support to participants via messaging (and less frequently, phone or video calls when requested) and reviews practice logs using a provider “dashboard” and electronic medical records that detail individual progress (including participant engagement and patient-reported outcomes to date). In total, therapists allocate 10 to 20 min (on average) per week per participant. Interaction between therapist and participant can be initiated via either party.

As a safety measure, therapists conduct a phone-based protocol assessment for any participants that show signs of mental deterioration during the intervention. In case of an emergency, such as active suicidal ideation with intent to act or the onset of psychotic symptoms, the intervention includes a written action plan for declining mental health, which all participants are required to review with their therapist prior to engaging with the intervention. In these situations, the care coordination-therapist team at Meru Health will help connect the patient to immediate and local care outside of the program, however, a psychiatrist employed by Meru Health is available for consultation in these situations as well.

Patients complete the MHP as a cohort of typically 8–15 other participants. As such, the group of patients can interact anonymously sharing their practice experiences, which provides and enables receipt of support and feedback on their experiences as they navigate the MHP. Free cross-talk between participants, however, is not allowed. Instead, participants can post anonymous reflections on practices and lessons to the chat discussion board, to which their therapist can respond freely, and to which other group members can respond with pre-written empathy statements and/or emoticons.

### 2.4 Measures

All measures examined in this study were collected in the MHP app before, during, and at end-of-treatment as well as at 3- and 6-months post program completion via email invitation.

#### 2.4.1 Primary outcome

The primary outcome of interest was change in depressive symptoms measured by the PHQ-9. Comprised of a list of nine depressive symptoms from the full PHQ with response options ranging from 0 (not at all) to 3 (nearly every day), the PHQ-9 is a widely used instrument used to screen for depression (Kroenke et al., 2001). Total scores range from 0 to 27, with scores of 10 or higher indicating a major depressive episode in validation studies (Löwe et al., 2004; McMillan et al., 2010). In general, scores of 5, 10, 15, and 20 indicate mild, moderate, moderately severe, and severe levels of depressive symptoms, respectively. The PHQ-9 has excellent internal consistency (Cronbach's α of 0.89 in primary care settings), and excellent test-retest reliability (Arroll et al., 2010). The assessments occurred in the MHP app at baseline and every 2 weeks during treatment, including the 12-week, end-of-treatment assessment and via email invitation at 3- and 6- months post-intervention.

#### 2.4.2 Other outcomes of interest

In addition to examining PHQ-9 depressive symptoms as a continuous measure, we also created two “response” variables based on the PHQ-9 reductions. First, we defined response as Kroenke et al. (2001) did in their original validation study that was further validated as a clinically significant improvement in symptoms in an additional comparison study (Kroenke et al., 2001): a 50% reduction in PHQ-9 score plus an end-of-treatment score of PHQ-9 < 10. We also tested a more liberal definition of response proposed by Lowe and colleagues that we have labelled as “clinically significant change” and calculated as a PHQ-9 score reduction of ≥5 (Löwe et al., 2004). We defined remission as having an end-of-treatment score of less than 5.

#### 2.4.3 Covariates

Various patient demographics and clinical characteristics collected at baseline as well as engagement metrics collected during the MHP were examined as correlates of depressive symptom response. These variables included age, gender, currently taking psychiatric medication, any lifetime suicide attempt, any lifetime psychiatric hospitalization, lifetime major traumatic event exposure, first episode versus recurrent major depressive disorder, proportion of total weekly introductions watched, number of minutes of in-program meditation, average number of days patient exchanged messages with therapist per week, and proportion of number of days active (e.g., any practices, watching content, or messaging) with the MHP. These covariates were selected based on prior studies showing significant associations with changes in depressive symptoms after treatment (Trivedi et al., 2006). Program completion was defined as watching at least half of the weekly psychoeducation video lessons during the program (e.g., at least at least 6 during the 12-week program), but program completion was not a requirement for study inclusion as our analyses were intent-to-treat (described below).

### 2.5 Sample size/power

An a priori sample size calculation was performed for comparing patient-reported depressive symptoms at baseline and follow-up time-points. 33 patients were required in the analysis given an alpha level of 0.05, a power of 0.8, and a medium effect size of 0.5. Thus, the present study, with 218 patients with baseline PHQ-9 ≥ 15 (moderately severe or severe depression) separated into two subgroups of 141 patients with PHQ-9 between 15 and 19, and 77 patients with PHQ-9 ≥ 20 (severe depression) was sufficiently powered to detect a medium effect in unadjusted analyses.

### 2.6 Statistical analysis

All statistical analyses were conducted with RStudio, Version 1.3.959 on patients with at least moderately severe depressive symptoms at baseline (PHQ-9 ≥ 15) and further subsetting to those with moderately-severe depressive symptoms (PHQ-9 between 15 and 19) and those with severe baseline depressive symptoms (PHQ-9 ≥ 20). Statistical significance was defined using 95% confidence intervals and *p*-values. Exploratory analyses including histograms as well as skew and kurtosis statistics were run for each variable to check for normality and any variable that then had a skew of ±2 was log transformed. Descriptive statistics (i.e., n and percentages or mean and standard deviations) were calculated for each patient demographic and clinical variable, engagement characteristic, and each outcome variable. Outcome measures were analysed using an intention-to-treat (ITT) analysis in which all participants with outcome measures at baseline were included, regardless of intervention engagement or attrition. 7.5% of data were missing from the final sample, ranging from 0% to 47.15% for PHQ-9 symptoms (note this study used ITT, so we included all treatment weeks 0–12 and 3- and 6-month follow-up data as missing for participants that dropped out; see Supplemental Figs. S1 and S2 for further details on variable missingness). To assess whether data were missing completely at random (MCAR) we performed a parametric (*p* < 0.001) test using the Naniar package (Tierney et al., 2021). Despite that data were not MCAR, based on recent recommendations (Enders, 2017; Matta et al., 2018), in order to account for missing data, we used multiple imputation (10 imputations) using the mice package (van Buuren, 2020) (see Supplemental Figures for missing data by variable and treatment week).

We used *t*-tests or chi-square tests to determine the bivariate (unadjusted) associations between each demographic, clinical, and engagement variable and response (50% reduction in PHQ-9 symptoms and end-of-treatment PHQ-9 < 10). We examined the impact of treatment week on PHQ-9 symptoms by conducting multilevel models (MLM) using the lme4 package (Bates, 2020) that nested week of treatment (Level-1) within individuals (Level-2). Fixed effects including covariates and treatment were examined at the level of participants (Level-2). These statistical approaches account for dependency within participants and provide unbiased estimates in the case of data missing completely at random, covariate-dependent missingness, and missingness at random (Matta et al., 2018), unlike methods such as repeated measures of analysis of variance (Raudenbush and Bryk, 2002). We used a data-driven model building approach to decide on which variables should be fixed vs random. For the linear model we examined the fixed linear association between treatment week and PHQ-9 symptoms allowing intercepts to vary. For the non-linear model we used treatment week centered and treatment week quadratic centered to examine both fixed linear and non-linear associations between treatment week and PHQ-9 symptoms allowing intercepts to vary. Model comparison indicated that the non-linear model fit best (delta AIC = 270, delta BIC = 265, *p* < 0.001). For each analysis, we report the *p* value and the Hedges' *g* effect size, calculated as the difference between the baseline and the assessment divided by the pooled and weighted standard deviation using the effectsize (Ben-Shachar et al., 2021) and ggstatsplot (Patil, 2020) packages (see supplementary materials for figures of effect sizes by symptom severity). An effect size of 0.5 and 0.8 are considered medium and large effect sizes, respectively (Hedges, 1981).

## 3 Results

### 3.1 Demographic and clinical characteristics

Table 1 presents demographic and clinical characteristics for patients with at least moderately severe depressive symptoms (PHQ-9 ≥ 15) and further subset by moderately-severe (PHQ-9 between 15 and 19) and severe depressive symptoms (PHQ-9 ≥ 20). On average, patients were 39 years of age and largely female with recurrent episodes of MDD. Over half reported currently taking psychiatric medication. In addition, over half reported exposure to a major trauma, while about 8% reported a lifetime suicide attempts and 8% reported a lifetime psychiatric hospitalization. Nearly 2 in 3 of the study sample met criteria for MHP completion. The mean baseline PHQ-9 was 18.60 (16.80 in the moderately severe and 22.0 in the severe depressive symptom group).

**Table 1:** Participant demographics.

Variable	N	Percentage	Mean (SD), range
Age	218		39.0 (11.2), 19–73
Sex			
Female	134	61.47%	
Male	39	17.89%	
Other	45	20.64%	
Baseline PHQ-9	218		18.60 (2.97)
Moderately severe group	141		16.80 (1.45)
Severe group	77		22.00 (1.80)
Lifetime recurrent MDD			
Yes	158	72.48%	
No	60	27.52%	
Taking psychiatric medication			
Yes	141	64.68%	
No	77	35.32%	
Lifetime suicide attempt			
Yes	18	8.26%	
No	200	91.74%	
Lifetime psychiatric hospitalization			
Yes	17	7.80%	
No	201	7.80%	
Lifetime history of major traumatic event exposure			
Yes	120	55.05%	
No	90	41.28%	
Unknown	8	3.67%	

### 3.2 Engagement

On average, participants watched about two-thirds of the weekly introductory videos and completed about 10 min of HRVB per week (Table 2). In addition, patients exchanged about 1 messages with their therapist per week and participated in the program just under an average of 3 days per week. There were no differences in engagement metrics between those with moderately-severe and severe depressive symptoms at baseline.

**Table 2:** Participant engagement characteristics.

Engagement characteristic	Complete sample (N = 218)	Patients with moderate or severe depression (PHQ-9 ≥ 15 and <20) at baseline (N = 141) Mean (sd)	Patients with severe depression (PHQ-9 ≥ 20) at baseline (N = 77) Mean (sd)	P-value
% of weekly introductions watched	67 (32)	67 (32)	66 (32)	0.80
Average number of meditation minutes per week	9.56 (11.2)	9.56 (11.0)	9.57 (11.6)	1.00
Average number of days patient exchanged at least one message with therapist per week	0.88 (0.76)	0.93 (0.81)	0.78 (0.65)	1.00
Average number of days active in program per week	2.71 (1.89)	2.69 (1.89)	2.73 (1.90)	0.90

sd: standard deviation, p-value represents difference between moderately severe and severe groups.

### 3.3 Changes in depressive symptoms

At end-of-treatment, patients experienced mean declines of 7.06 and 10.6 PHQ-9 points among those with baseline PHQ-9 between 15 and 19 and PHQ-9 ≥ 20, respectively (a 41.80% and 47.30% reduction in symptoms in the two subsamples; Table 3). These mean changes correspond to a Hedges' *g* effect size of 1.64 (95%CI = 1.44–1.85) for the overall sample (g = 1.62 for those with moderately-severe and 1.92 for those with severe depressive symptoms at baseline; Supplemental Figs. S3–S5). A majority (71.6% and 90.9% in the moderately severe and severe subsamples, respectively) experienced a clinically significant decline of 5 or more PHQ-9 points between baseline and end-of-treatment assessments. Just under a third of those with at least moderately severe depressive symptoms at baseline responded to the MHP intervention (defined as at least a 50% reduction in symptoms plus an end PHQ-9 score of less than 10). Participants with severe depressive symptoms at baseline had greater mean declines of symptoms, a larger proportion of those with a clinically significant decline defined as at least a 5-point decline and with at least a 50% decline in depressive symptoms than participants with moderately-severe depressive symptoms at baseline.

**Table 3:** Depression outcome characteristics of meru health patients with moderate or severe depression at start of program.

Outcome characteristic	Complete sample (N = 218)	Patients with moderate or severe depression (PHQ-9 ≥15) at baseline who completed the intervention (N = 141) n (%)	Patients with severe depression (PHQ-9 ≥20) at baseline who completed the intervention (N = 77) n (%)	P-value
At end-of-program				
PHQ-9 (mean, sd)	10.3 (4.48)	9.71 (4.18)	11.40 (4.81)	**0.008**
PHQ reduction (mean, sd)	−8.30 (5.03)	−7.06 (4.33)	−10.60 (5.44)	**<0.001**
PHQ reduction (mean % reduction, sd)	−43.80 (24.50)	−41.80 (25.10)	−47.30 (23.20)	0.100
5+ point decline in PHQ-9				**0.002**
Yes	171 (78.4%)	101 (71.6%)	70 (90.9%)	
No	37 (21.6%)	40 (28.4%)	7 (9.09%)	
50% + decline in PHQ-9				**0.020**
Yes	87 (39.9%)	48 (34.0%)	39 (50.6%)	
No	131 (60.1%)	93 (66.0%)	38 (49.4%)	
Response^a^				0.600
Yes	71 (32.6%)	48 (34.0%)	23 (29.9%)	
No	147 (67.4%)	93 (66.0%)	54 (70.1%)	
Remission^b^				0.300
Yes	31 (14.2%)	23 (16.3%)	8 (10.4%)	
No	187 (85.8%)	118 (83.7%)	69 (89.6%)	

PHQ-9: Patient Health Questionnaire-9 item version; sd: standard deviation.

Bold indicates significance of p < 0.05

a At least 50% reduction in PHQ-9 AND end PHQ-9 score < 10.

b End PHQ-9 score of <5.

**Fig. 1:**
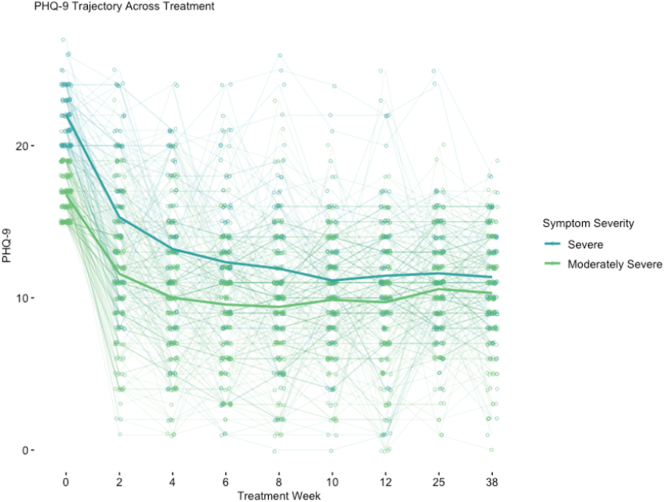
Depressive symptom trajectory from baseline to 6-months post-treatment.

In adjusted models, patients continued to experience significant decreases in PHQ-9 scores (β = −0.215, *p* < 0.001) throughout treatment and the follow-up assessment points (Table 4 and Fig. 1). In addition, there was a significant quadratic effect (β = 0.532, p < 0.001) where participants experienced faster decreases in the early weeks of the MHP that tapered off towards the end of treatment and follow-up. Individual participant depressive symptom trajectories are displayed in Supplemental Fig. S6.

**Table 4:** Adjusted models of depressive symptoms from baseline to 6-months post-treatment.

Predictors	Linear model				Quadratic model			
	Standardized beta	Standardized std. error	Standardized CI	P-value	Standardized beta	Standardized std. error	Standardized CI	P-value
(Intercept)	0.068	0.097	−0.123–0.259	**<0.001**	0.066	0.097	−0.125–0.256	**<0.001**
Treatment week	−0.215	0.020	−0.253 to −0.177	**<0.001**				
Treatment week (centered)					−0.647	0.031	−0.708 to −0.587	**<0.001**
Treatment week quadratic (centered)					0.532	0.031	0.472–0.593	**<0.001**
Sex - male	0.092	0.110	−0.123–0.307	0.402	0.093	0.110	−0.122–0.308	0.396
Sex - other	0.048	0.103	−0.155–0.250	0.644	0.046	0.103	−0.156–0.247	0.657
Age	−0.024	0.040	−0.102–0.055	0.556	−0.024	0.040	−0.103–0.055	0.552
Proportion of introduction videos watches	−0.110	0.082	−0.270–0.051	0.180	−0.110	0.082	−0.271–0.050	0.177
Proportion of days active	−0.121	0.118	−0.352–0.110	0.305	−0.121	0.118	−0.352–0.110	0.306
Recurrent MDD	0.034	0.091	−0.144–0.213	0.707	0.033	0.091	−0.145–0.212	0.717
History of psychiatric hospitalization	0.245	0.167	−0.082–0.572	0.143	0.245	0.167	−0.082–0.572	0.142
Suicide attempt history	−0.158	0.158	−0.467–0.151	0.315	−0.158	0.158	−0.467–0.151	0.317
Major trauma history - unknown	0.384	0.205	−0.019–0.786	0.062	0.384	0.205	−0.019–0.786	0.062
Major trauma history - yes	0.036	0.083	−0.127–0.199	0.664	0.037	0.083	−0.127–0.200	0.661
Psychotropic medication	−0.243	0.080	−0.399 to −0.087	**0.002**	−0.238	0.078	−0.391 to −0.086	**0.002**
Meditation minutes per week	0.068	0.082	−0.093–0.229	0.405	0.068	0.082	−0.092–0.229	0.404
Average text messages to therapist per week	0.029	0.053	−0.074–0.133	0.579	0.029	0.053	−0.074–0.133	0.577


Random effects		
σ^2^	18.04	15.26
τ_00_	5.56 _userID_	5.87 _userID_
ICC	0.24	0.28
N	203 _userID_	203 _userID_
Observations	1827	1827
Marginal R^2^/conditional R^2^	0.094/0.308	0.189/0.414

Bold indicates significance of p < 0.05

### 3.4 Correlates of response and decreases in depressive symptoms

The only significant correlate of response in bivariate analyses was whether the participant reported taking psychotropic medication upon MHP entry, with those reporting medication having greater decreases in depressive symptoms over the study period than those not reporting medication. None of the other demographic, engagement, or clinical characteristics in the model were associated with depressive symptom changes over the course of the study.

## 4 Discussion

This study shows preliminary feasibility and improvements in depressive symptoms sustained up to 6-months post-treatment among patients with moderately-severe to severe depressive symptoms who participate in the MHP, a therapist-supported, evidence-based program delivered via a smartphone app. Patients displayed moderate levels of engagement with the program, with average weekly participation in the program nearly 3 days with about 10 min of HRVB practice per week. Severity of depressive symptoms was not related to engagement. The MHP completion rate of about two-thirds in this participant sample with at least moderately severe depressive symptoms is in the range of meta-analyses reporting on completion rates among all depressed psychotherapy patients, regardless of symptom severity. In addition, many of the studies included in these meta-analyses that quantified attendance of sessions in the drop-out definition had lower thresholds for program completion (e.g., 3 sessions), which would make it easier for patients to meet that completion criteria. Finally, each of the participants included in our study analysis were enrolled either during or after the declaration of the COVID-19 pandemic, so likely were experiencing significant challenges related to social isolation, economic implications, and/or fear of contracting the virus itself that may have played a role in having the ability to adhere to treatment recommendations. Regardless, a more stringent definition of completion, combined with a comparable completion rate, indicates that patients with moderately severe to severe depression adhered and responded particularly well to the MHP.

Reductions in symptoms were also significant for this patient population. Patients with at least moderately severe depressive symptoms at baseline also experienced significant reductions in depressive symptoms at end-of treatment (mean PHQ-9 reduction = 8.3, Hedges' *g* = 1.64). This finding suggests that, even with at least moderately severe levels of depressive symptoms at baseline, MHP patients were able to engage and complete the MHP.

In terms of other quantifications of outcomes, a large majority of participants experienced a clinically significant change in depressive symptoms, defined as having at least a 5-point decline during treatment. In fact, nearly all (90.9%) patients with severe depression at baseline had at least a 5-point decline. In terms of response defined stringently as having at least a 50% decline in depressive symptoms AND having an end score of less than 10 on the PHQ-9, about having about a third of patients with at least moderately severe depressive symptoms at baseline (i.e., experienced at least a 50% reduction in PHQ-9 score and had a post-program PHQ-9 score lower than 10). Each of these effect sizes are similar to those found in prior studies that have tested other types of interventions for patients with higher severity levels of depression (DeRubeis et al., 2005), including the “real world” STAR-D trial of flexible citalopram dosing that defined response as a 50% reduction in symptoms, which found rates of 36% after 8 weeks and 43% after 12 weeks (Trivedi et al., 2006). Findings from the present study also mirror those from a trial testing an internet cognitive behavioral therapy intervention that included patients with moderately-severe and severe levels of depressive symptoms, which found significant reductions in depressive symptoms sustained up to 11 months post-treatment (Lindegaard et al., 2019) and an observational study of a national digital mental health service in Australia that found similar effect sizes for their depressed patients (Titov et al., 2020). Finally, the current study's finding of MHP having efficacy among those with moderately severe and severe depressive symptoms is similar to that determined by a study that found significant declines in depressive symptoms among patients who had a schizophrenia spectrum disorder, bipolar disorder, or major depressive disorders who each had at least moderately severe levels of depressive symptoms (Ben-Zeev et al., 2019).

It is important to note that the smartphone intervention tested, the MHP overcomes several of the barriers that typically keep patients from starting and continuing care. Pharmacological treatments often have associated side effects and can take four to six weeks until its effects are realized. More intensive treatments like ECT, brain stimulation therapies, and anaesthetics like ketamine often work quickly but effects are not often sustained (Ionescu and Papakostas, 2017). The resulting instances of relapse can leave the patient in a similar or even worse situation than the one experienced when presenting for treatment—depressed and, perhaps even more, hopeless. In addition to low side effect profiles and apparent improvement in outcomes, the current MHP intervention also addresses several common barriers to seeking mental health care, including scarcity of trained clinicians, access in remote areas, time for appointments, and stigma (SAMHSA, 2019). The MHP does not require the patient to have access to transportation to a medical facility or a visit with a mental health care provider, which are currently sparse in the U.S (Health, 2017). In addition, the smartphone intervention tested can be utilized at the discretion of the user, which reduces stigma and provides flexibility in when treatment can occur. Thus, the smartphone intervention tested might be a desirable and effective alternative to other types of care currently used to treat more severe forms of depression.

The study findings should be interpreted in light of several limitations, most of which relate to the use of real-world data collected as part of continuous improvement of a mHealth intervention. First, we examined only levels of depressive symptoms and did not have impairment or any other clinical diagnostic data to examine. The findings therefore do not directly relate to patients with major depressive disorder with severe impairment, for example. Indeed, the rates of lifetime psychiatric hospitalizations (7.80%) and lifetime suicide attempts (8.26%) of the current sample of participants with at least moderately severe depressive symptoms is certainly higher than those of the general population but lower than similar studies like that published by DeRubeis et al. (2005) where 12% of patients with moderate to severe clinical depression (rather than depressive symptoms) had a psychiatric hospitalization. Second, this analysis of clinical data collected in a real-world setting precluded the comparison of findings to a control group. Because depressive symptoms tend to wax and wane, we cannot be sure that patients in our study would not have improved on their own without treatment.

Depression currently ranks second among all diseases and injuries in the U.S. as a cause of disability (Murray et al., 2013). Severe depression, in particular, can be life threatening due to increased risk of suicide (Wulsin et al., 1999). Alternative treatments are urgently needed, specifically those that improve access to care and reduce the harms of treatment. This real-world study indicates the potential efficacy of one of these alternative treatments, the MHP, to treat patients with moderately severe to severe depressive symptoms. One potential reason for the effectiveness of the MHP may be that therapists overseeing the program provide a type of continuous care for patients by first establishing rapport and conveying empathy and then developing a therapeutic alliance and instilling hope in patients, which increase program engagement and, consequently, the likelihood of symptoms improvement and recovery (Elliott et al., 2018; Krupnick et al., 2006; Kvedar et al., 2014; Mullins et al., 2012). Future randomized trials are warranted to test the MHP either as a scalable solution or as an integrated part of existing treatment types for patients with more severe symptoms of depression. In addition, study of provider acceptability of using the MHP to treat patients with more severe forms of depression is also needed, particularly in light of prior studies that have found providers to see digital health treatments as suitable to treat more mild forms of depressive symptoms (Topooco et al., 2017).

## Funding

This research did not receive any specific grant from funding agencies in the public, commercial, or not-for-profit sectors.

## Declaration of competing interest

Dr. Forman-Hoffman is employed as the Chief Research Officer at Meru Health, Inc., receives salary from the company and owns options of the company. Dr. Nelson is an employee of Meru Health, Inc. Mr. Ranta serves as the Chief Executive Officer (CEO) at Meru Health, Inc., owns a large share of stocks, and raises salary from the company. Dr. Hilgert is employed as Director of Therapy Development and Clinical Operations at Meru Health Inc., receives salary from the company, and owns stocks and options of the company. Mr. Nazander serves as the Chief Technology Office (CTO) at Meru Health Inc., owns a large share of stocks, and raises salary from the company. Dr. de Quevedo has no competing financial interests.

## Appendix A Supplementary data

Supplementary figures Image 1
