# Supplementary material for: Significant reduction in depressive symptoms among patients with moderately-severe to severe depressive symptoms after participation in a therapist-supported, evidence-based mobile health program delivered via a smartphone app
Source: Internet Interv. 2021 Jun 17;25:100408. doi: 10.1016/j.invent.2021.100408 (PMC8350582; doi:10.1016/j.invent.2021.100408)
Supplement: Supplementary file 1 — Supplementary figures [file mmc1.docx]

**Supplementary Materials**

Table of Contents

Missing Data 2

Supplemental Figure S1. Missing Data Overall 2

Supplemental Figure S2. Missing Data by Treatment Week and Follow-Up Assessment 3

Individual Depression Trajectories 4

Supplemental Figure S3. Individual Participant Depressive Symptom Trajectories by Treatment Week and Follow-Up Assessment 4

Effect Sizes by Symptom Severity 5

Supplemental Figure S4. Effect Size for All Participants with at Least Moderately Severe Depressive Symptoms 5

Supplemental Figure S5. Effect Size for Participants with Moderately-Severe Depressive Symptoms 6

Supplemental Figure S6. Effect Size for Participants with Severe Depressive Symptoms 7

# **Missing Data**

## **Supplemental Figure S1. Missing Data Overall**

**
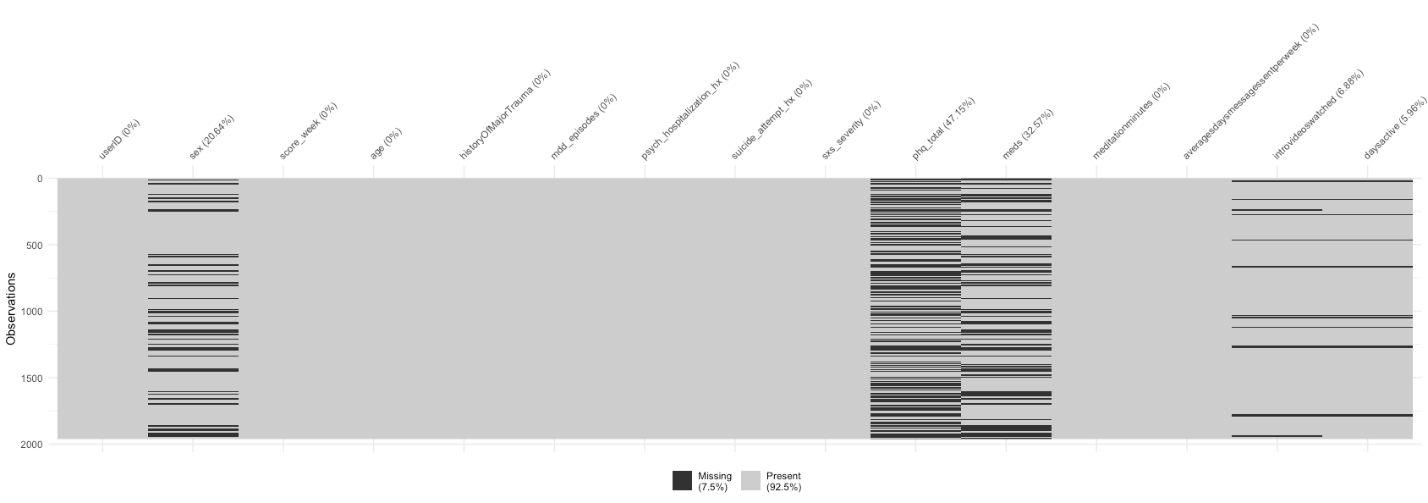
**

## **Supplemental Figure S2. Missing Data by Treatment Week and Follow-Up Assessment**

**
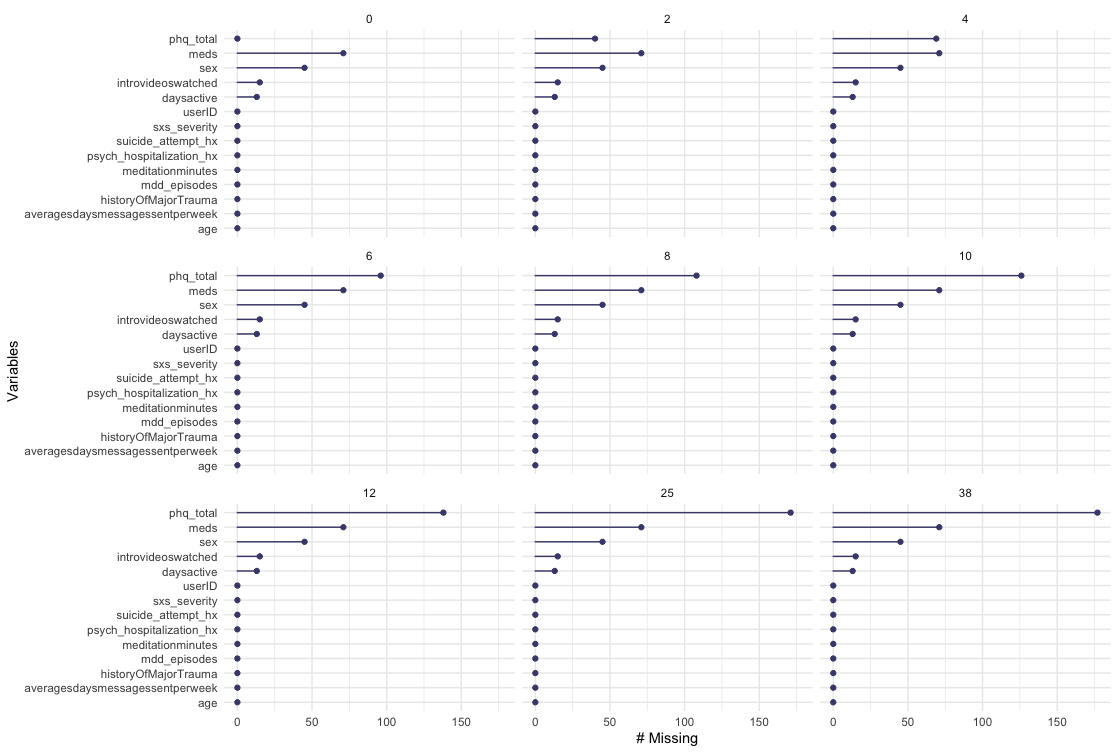
**

# **Individual Depression Trajectories**

## **Supplemental Figure S3. Individual Participant Depressive Symptom Trajectories by Treatment Week and Follow-Up Assessment**

**
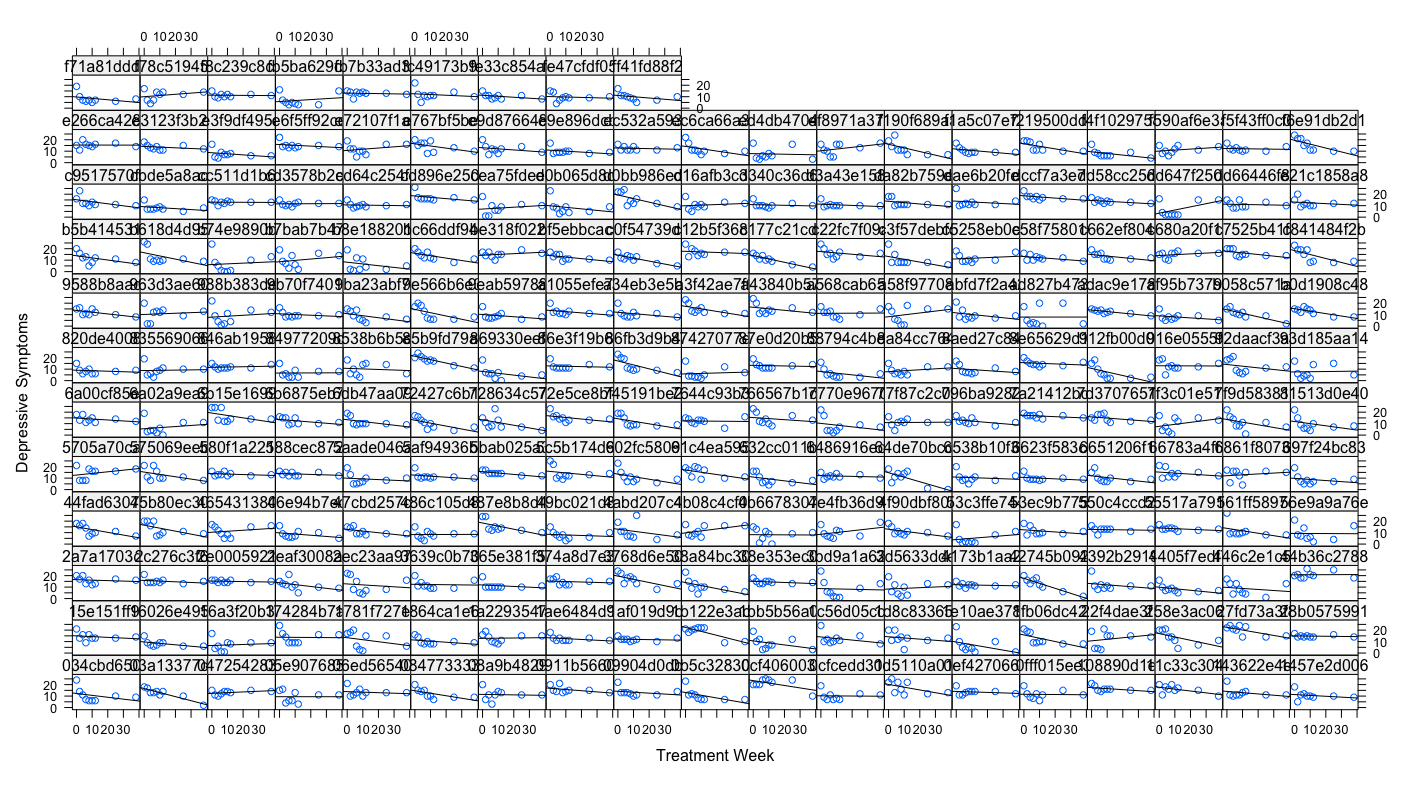
**

# **Effect Sizes by Symptom Severity**

## **Supplemental Figure S4. Effect Size for All Participants with at Least Moderately Severe Depressive Symptoms**

**
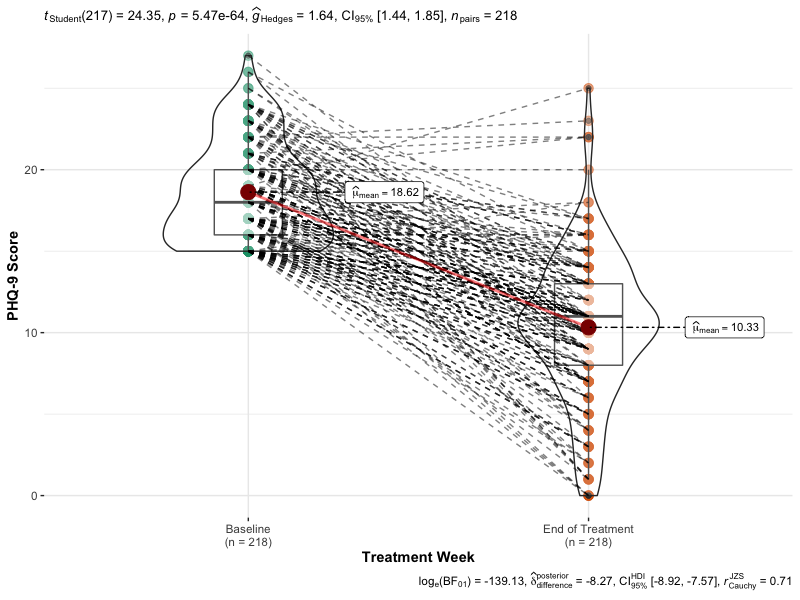
**

## **Supplemental Figure S5. Effect Size for Participants with Moderately-Severe Depressive Symptoms**

**
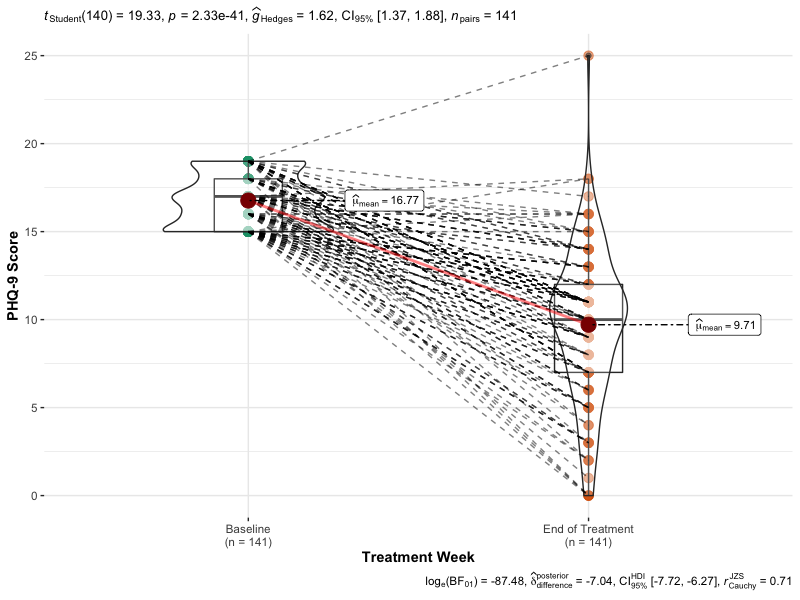
**

## **Supplemental Figure S6. Effect Size for Participants with Severe Depressive Symptoms**

**
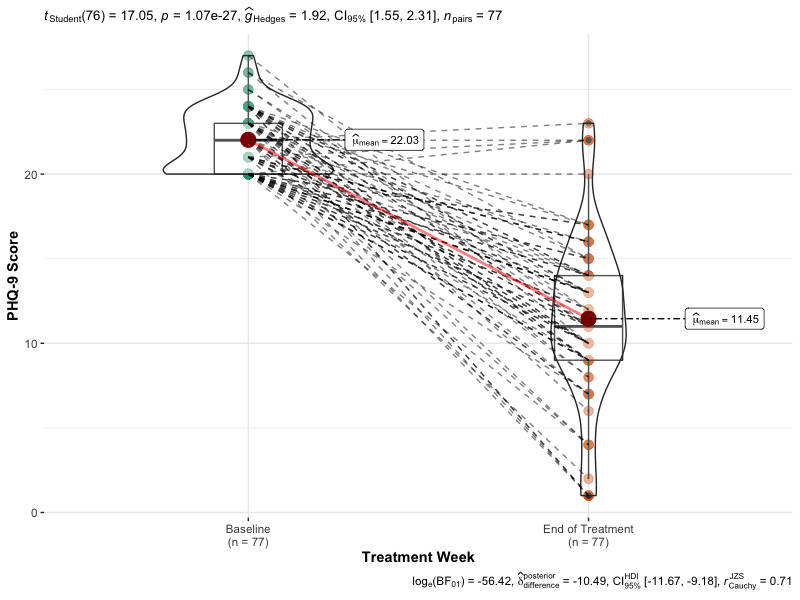
**
